# Position Impact of Hydroxy Groups on Spectral, Acid–Base Profiles and DNA Interactions of Several Monohydroxy Flavanones

**DOI:** 10.3390/molecules24173049

**Published:** 2019-08-22

**Authors:** Elżbieta Łodyga-Chruścińska, Agnieszka Kowalska-Baron, Paulina Błazińska, Maria Pilo, Antonio Zucca, Violetta M. Korolevich, Vitali T. Cheshchevik

**Affiliations:** 1 Faculty of Biotechnology and Food Sciences, Lodz University of Technology, Stefanowskiego Street 4/10, 90-924 Lodz, Poland; 2 Dipartimento di Chimica e Farmacia, Università di Sassari, via Vienna 2, I-07100 Sassari, Italy; 3 Department of Biotechnology, Polessky State University, Str. Dnieper flotilla 23, 225710 Pinsk, Belarus

**Keywords:** monohydroxy flavanones, dissociation constants, DNA interactions, structure–activity relationship

## Abstract

Structure-related biological activities of flavanones are still considered largely unexplored. Since they exhibit various medicinal activities, it is intriguing to enter deeper into their chemical structures, electronic transitions or interactions with some biomolecules in order to find properties that allow us to better understand their effects. Little information is available on biological activity of flavanone and its monohydroxy derivatives in relation to their physicochemical properties as spectral profiles, existence of protonated/deprotonated species under pH changes or interaction with Calf Thymus DNA. We devoted this work to research demonstrating differences in the physicochemical properties of the four flavanones: flavanone, 2′-hydroxyflavanone, 6-hydroxyflavanone and 7-hydroxyflavanone and linking them to their biological activity. Potentiometric titration, UV–Vis spectroscopy were used to investigate influence of pH on acid–base and spectral profiles and to propose the mode of interaction with DNA. Cyclic voltammetry was applied to evaluate antioxidant potentiality and additionally, theoretical DFT(B3LYP) method to disclose electronic structure and properties of the compounds. Molecular geometries, proton affinities and pK_a_ values have been determined. According to computational and cyclic voltammetry results we could predict higher antioxidant activity of 6-hydroxyflavanone with respect to other compounds. The values of K_b_ intrinsic binding constants of the flavanones indicated weak interactions with DNA. Structure–activity relationships observed for antioxidant activity and DNA interactions suggest that 6-hydroxyflavanone can protect DNA against oxidative damage most effectively than flavanone, 2′-hydroxyflavanone or 7-hydroxyflavanone.

## 1. Introduction

Flavonoids belong to a large group of polyphenolic compounds with the structure of benzo-γ-piron and are commonly found in plants. They are hydroxylated phenolic substances and are known to be synthesized by plants in response to microbial infection [1]. Many flavonoid structures display a variety of biochemical properties, including estrogenic, antioxidant, antiviral, antibacterial, antiobesity, and anticancer activities. The diverse pharmacological activities of flavonoids have drawn considerable attention for their use in personal health care [2]. With the publicity given to the beneficial effects, the consumption of dietary supplements containing flavonoids has increased significantly. It has been pointed out that the excess use of these compounds could have drastic effects, as high concentrations of flavonoids may act as mutagens, pro-oxidants and inhibitors of hormone metabolizing enzymes. Their chemical nature depends on their structural class, degree of hydroxylation and polymerization or other substitutions and conjugations. It affects their bioavailability and pharmacological action. Flavanone is the precursor to all flavonoid structures. Flavanones, which fall under a class of flavonoids, have important antioxidant properties [3,4] and interesting biological activities [5,6]. It has been indicated that flavanones with none or single OH group has anti-proliferation potential on colorectal carcinoma cells and mouse fibroblast NIH3T3 cells [7,8]. 6-Hydroxyflavanone is one of the members of above class of flavanoids. It is a monohydroxyflavanone which has hydroxy group at its 6th position. It has been reported [9] that flavanone with the only hydroxylation at C6 has a significant cytotoxic effect in human leukemia HL-60 cells accompanied by the occurrence of apoptotic bodies, and hypodiploid cells, characteristics of apoptosis. In turn 2′-hydroxyflavanone, a nontoxic natural flavonoid has exhibited pleiotropic anticancer effects in many cancer types, including prostate and breast cancers [10,11]. On the other side, it has been found that 7-hydroxyflavanone has weak effect on inhibiting MCF-7 cell proliferation [12] and low cytotoxic effect on acute lymphoblastic leukemia (ALL) and chronic myeloid leukemia (CML) cell lines [13].

The above mentioned interesting pharmacological potential of flavanone and its monohydroxy derivatives prompted us to investigate these chemical compounds. Structure-related biological activities of flavanones are still considered unexplained. Since they exhibit various medicinal activities, it is intriguing to enter deeper into their chemical structures, electronic transitions or interactions with some biomolecules in order to find properties that allow us to better understand their effects. Such a knowledge will create the possibility of more conscious and purposeful use of these compounds both in medicine and in dietary supplements. Little information is available on biological activity of flavanone and its monohydroxy derivatives in relation to their physicochemical properties such as spectral profiles, existence of protonated/deprotonated species under pH changes or interaction with Calf Thymus DNA (CT DNA). Studies in solution are indispensable, because biologically active substances act in the cell compartments with different water contents, and therefore, the information about the acid–base properties give an idea of which chemical forms e.g., protonated/deprotonated of biomolecule may be involved in biochemical processes in the cells. We devoted this work to research aimed at demonstrating differences in the physicochemical properties of the four flavanones and linking them to their biological activity.

Recently, computational simulations are more and more frequently applied in the studies on electronic structures and properties of biologically important molecules. Theoretical studies may provide very useful and, in many cases unavailable experimentally, information about the structure and properties of biomolecules. Among various theoretical methods, density functional theory (DFT) approach has been extensively used in the prediction of structure-related properties of flavanones and structurally similar compounds [14,15,16]. In particular it is possible to conclude about antioxidant activity of flavanones based on the analysis of the DFT predicted frontier orbitals and the DFT calculated quantum chemical descriptors [14].

UV–Vis spectra of medium-size molecules such as flavanones and structurally related compounds are usually well predicted using the time-dependent TD DFT formalism [14,17,18]. Within the framework of the TD DFT theory, the time-dependent oscillating electric field is incorporated in the ground state structure and excitation energies, oscillator strengths and transitions vectors can be determined from the linear response [19]. DFT and TD DFT methods have been usually shown to provide reasonable and consistent with experimental data results with relatively low computational costs. Previously performed theoretical studies on phenolic compounds with the hybrid Becke’s three parameters with Lee–Yang-Parr (B3LYP) functional [20,21,22] give results consistent with experimental data [23,24,25]. The choice of the applied exchange-correlation functional and the utilized basis set is usually the compromise between the time of computations and the quality of the obtained results. Although previously published studies [18] showed that application of 6-31G (d,p) basis set usually gives reasonable molecular geometries of chalcone molecules, the incorporation of diffuse and polarization functions is mandatory for UV spectral studies, especially for chromophores with extended π electrons [26]. 

It is well known that the type of solvent may affect UV–Vis spectra of chromophores, therefore, the incorporation of solvent effects is very important in the theoretical prediction of the absorption spectra. Polarizable Continuum Model (PCM) is the most frequently incorporated solvation model in theoretical studies of flavanones and chalcones structurally similar to the compounds under study [14,15]. The PCM model [27,28] is based on the assumption that solute is embedded in a shape-adapted cavity within solvent modelled as a dielectric continuum of defined dielectric constant. 

To the best of our knowledge only few previously published studies have been devoted to the structure and properties of the studied hydroxyflavanones and their corresponding chalcones. Wróblewski and et. [15] have applied both experimental (absorption and fluorescence spectroscopy) and theoretical (TD DFT(PCM)) methods to describe the photophysical characteristic of 7-HF in methanol, ethanol and acetonitrile. 

The structure and spectral characteristic of 2′hydroxychalcone and its derivatives with different alkyloxy groups at position 4′ were previously extensively investigated by Serdiuk et al. [16]. The authors concluded that generally both in crystals and solution there is a little impact of substituents on absorption spectra of chalcones. The authors revealed that 2′hydroxychalcones form crystal lattices with different packing patterns. Hydroxychalcones are planar in gas and crystalline phase and they do not change the conformation upon excitation. The authors proposed that in liquid media several processes for the excited state deactivation such as isomerization in the S_1_ state (geometrical changes), intersystem crossing and conical intersection are possible. The authors concluded that molecular conformation is the key factor determining the fluorescent properties of the phototautomer keto species formed by the excited state intramolecular proton transfer (ESIPT). The ESIPT process in 2′ hydroxychalcones, which is facilitated by the presence of hydrogen bond between hydroxy and carbonyl groups, was also studied previously [29]. 

The aims of this work were to investigate influence of pH on acid–base and spectral profiles of flavanone (F), 2′-hydroxyflavanone (2′HF), 6-hydroxyflavanone (6HF) and 7-hydroxyflavanone (7HF) and to reveal their interactions with CT DNA. Potentiometric titration, UV–Vis spectroscopy were used to characterize physicochemical properties of these flavanones and to propose the mode of interaction with CT DNA. Cyclic voltammetry was applied to evaluate antioxidant potentiality of studied flavanones. Additionally, a theoretical DFT (B3LYP) method has been used to disclose electronic structure and properties of the compounds. In particular molecular geometries, proton affinities and pKa values have been determined. Moreover, the time dependent DFT (TD DFT(B3LYP)) calculations have been performed on the optimized geometries to provide insight into the energies and nature of the electronic transitions which contribute to the absorption spectra of F, 2′HF, 6HF and 7HF.

## 2. Results and Discussion

### 2.1. Spectral Profiles of Flavanones

**Scheme 1:**
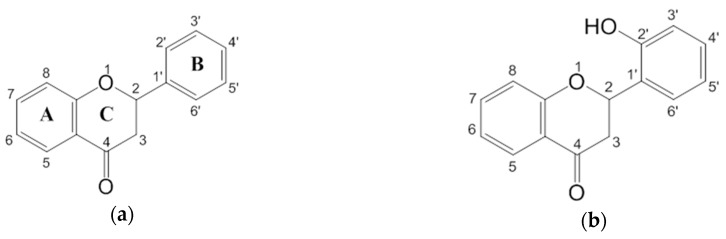

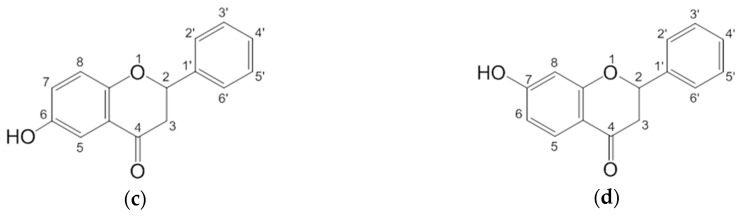
Chemical structures of flavanones: (**a**) flavanone; (**b**) 2′-hydroxyflavanone; (**c**) 6-hydroxyflavanone; (**d**) 7-hydroxyflavanone (ChemDraw).

Chemical structures of flavanones used in this study are illustrated in Scheme 1. 

These compounds shared the similar basic structure but they differ by hydroxylation pattern. Flavanone is one major component of flavonoids that is composed of two benzene rings (A and B) linked through a heterocyclic pyran ring (C) in the middle. The 6-OH flavanone (6HF), 7-OH flavanone (7HF) and 2′-OH flavanone (2′HF) contain an OH group on one of benzene rings. Evaluation of UV–Vis absorption spectra of polyphenols is of great importance to (1) predict optical spectra of new compounds, (2) understand colour modulation in fruit and flowers, (3) estimate the capacity of compounds to act as UV filters and (4) understand the capacity of compounds to be chemically transformed by UV light induction. The UV/Vis spectra of polyphenols are generally attributed to electronic transitions between π-type molecular orbitals, which are more or less extended over the molecular backbone, depending on the polyphenol subclass. In flavanones, due to the absence of the double bond between C2 and C3, electron delocalization is broken and transition of HOMO→LUMO orbitals becomes completely forbidden, due to orbital separation [14]. It leads to substantially reducing the conjugation, as compare to flavonols or flavones. Therefore, flavanones are colorless, while flavones and flavonols are yellow/-ish. It is worth noting that the absence of the 2,3-double bond brings about the existence of two stereoisomers for flavanones. However, the UV/Vis spectra of these isomers are very similar and it is difficult to distinguish them from each other.

**Figure 1:**
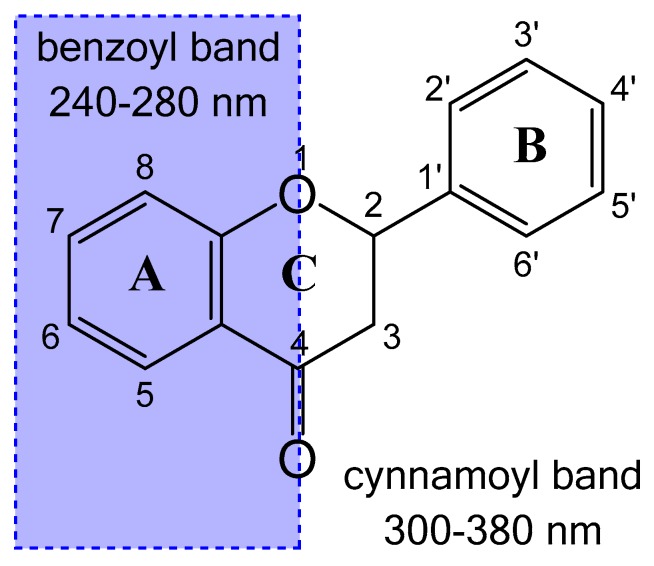
Benzoyl unit responsible for the band II and cinnamoyl unit responsible for the band I in absorption spectra of flavanones (ChemDraw).

The absorption spectra of 2 × 10^−5^ M solutions of flavanone, 6-hydroxyflavanone, 7-hydroxyflavone and 2′-hydroxyflavanone were obtained at different pH in aqueous solution containing 6.25 × 10^−3^ M HNO_3_ or HCl, 0.1 M NaCl and 0.5% of DMSO (Figure S1). The flavanones differed in electronic structure under pH changing. Hydroxide ions were added into solution and these ions effected the molecular structure of the compounds. Protonated species released protons. The UV spectra of flavanones show usually two strong absorption bands commonly referred to as band I (300–380 nm) and band II (240–280 nm) [30,31]. Band I is associated with the presence of a B-ring cinnamoyl system. Band II absorption is due to A-ring benzoyl system (Figure 1).

**Figure 2:**
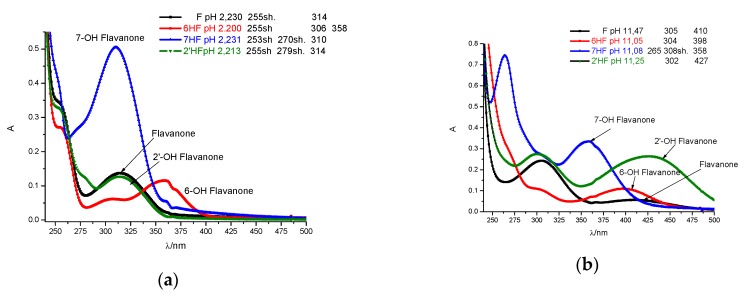
Comparison of absorption spectra of F, 6HF, 7HF and 2′HF in acidic (**a**) and alkaline (**b**) ranges. (Origin(Pro) software using the original files from the instrument: Perkin-Elmer Lambda 11 spectrophotometer).

Substitutions on the A or B ring may produce hypsochromic or bathochromic shifts of the absorptions, which are useful for clarifying structures [32]. The spectra in Figure 2 and Figure S1 indicate different behavior of F, 6HF, 7HF and 2′HF although they share a similar basic structure, but their different hydroxylation pattern has an impact on spectral profiles under the pH changes. 

The clear differences in the spectral profile of flavanones as a function of pH are attributed to the formation of different protolytic species forming especially in strongly basic solution whose light absorbing characteristics differ from each other. Flavanones studied exhibit a band I situated at 358 nm for 6HF, 314 nm for F and 2′HF, 310 nm for 7HF. The all flavanones disclose shoulders at around 255 nm of band II in acidic pH while in basic region only 7HF reveals the presence of this band located at around λ_max_ 265 nm (Figure 2). The long wavelength absorption band is assigned to the HOMO-3→LUMO transition in which the charge densities of both orbitals are strongly localized on ring A [14,33]. This plays an important role in shifting the absorption maxima of flavanones. An exceptional behavior of 7HF can indicate different mechanism associated with deprotonation of 7–OH group with comparison to 6–OH or 2′–OH groups in basic pH. On the other hand, another exceptional behavior but at acidic pH is observed for 6HF. The spectra of this flavanone recorded in the HNO_3_ and HCl media differ from each other in comparison to the spectra of the F, 7HF or 2′HF. Bathochromic shift of band I and its splitting in HNO_3_ solution with respect to the HCl-NaCl medium is clearly seen in the spectrum of 6HF. This may be due to the fact that the structure of 6HF is highly sensitive to electronic state and, very likely, the charge densities of orbitals localized on A-ring are more susceptible to changes in the surrounding environment than in the case of other flavanones. Such a spectral profile of 6HF indicates an interaction of the compound with protic solvent which originates from intermolecular hydrogen bonding [34]. Red shift in absorption maximum may indicate stronger influence of intermolecular hydrogen bond formation to stabilize molecular ground state [35]. It is thought to be a key feature of polar protic solvents [36]. Hence, one can infer that 6HF is more sensitive to the microenvironmental surroundings compared to other flavanones.

**Figure 3:**
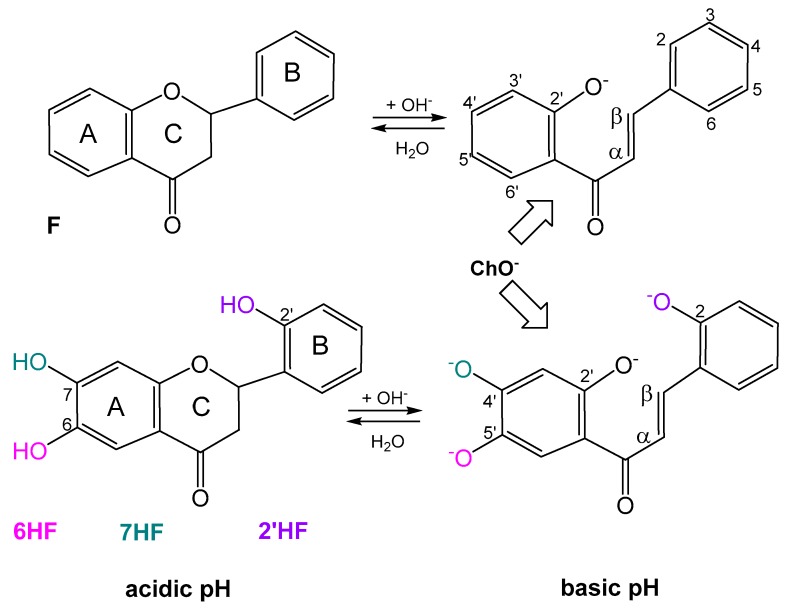
Overview of formation of chalcones for: F, 6HF, 2′HF and 7HF. Different colors refer to: 6HF (**magenta**); 2′HF (**violet**) and 7HF (**green**), respectively. (ChemDraw).

All flavanones form chalcones in alkaline medium. Chalcones or benzylideneacetophenone are the important constituents of natural sources. The structure of parent molecule of chalcones consists of two phenyl rings (A and B) and one α, β unsaturated double bond (Figure 3). Structures of flavanones especially the position of B ring have an impact on chalcone formation. Spectra of flavanones studied can indicate formation of chalcones in F, 6HF, 7HF and 2′HF systems. Certain amounts of both isomers, i.e., flavanones and their hydroxychalcones (ChOH) are measurable in final equilibrium at pH above 11. (Figure 2, see also Figure 11 in Section 2.4). The susceptibility of these compounds to chalcone formation may be visualized as the result of increased acidity of the hydrogen atom alpha to the carbonyl group coupled with ionization of the hydroxyl group. Chalconate anion (ChO-) prevails, accordingly to a proton-transfer reaction: F° + OH^−^ ⇆ ChO^−^ + H_2_O in excess of strong base (NaOH) [37] (Figure 3).

When the chalcone structural features are formed the spectra of F, 6HF and 2′HF show a broad maximum in the region of 398–427 nm (Figure 2b) similar to the most of flavanones [32]. The spectrum of 7HF exhibits a completely different spectral profile in basic pH (strong bands at 265 and 358 nm) but similar to other 7-hydroxyflavanones as for example liquiritigenin [30]. Taking into account our results and literature data one can conclude that all flavanones studied are able to form chalcones in alkaline medium.

### 2.2. Theoretical Calculations

#### 2.2.1. The Electronic Structures of the Compounds Studied Predicted by the DFT(B3LYP)/6-31G(d,p)/PCM Method

**Figure 4:**
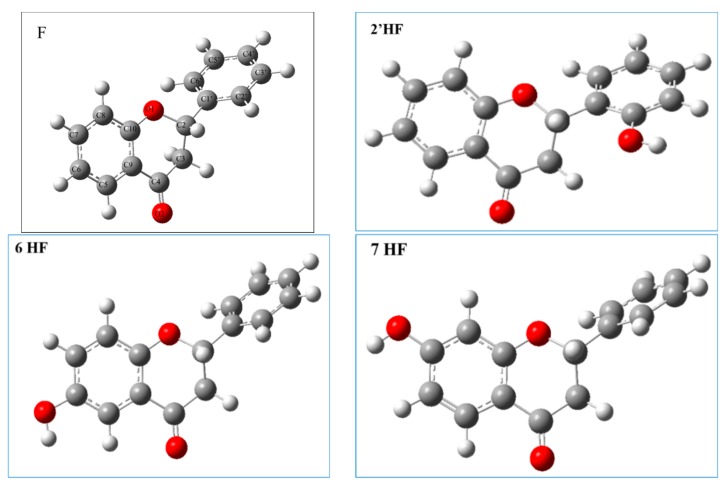
The DFT(B3LYP)/6-31G(d,p)/PCM optimized geometries of the 2S-flavanones: F, 2′HF, 6HF and 7HF. The atom numbering is presented for F; the same is for 2′HF, 6HF and 7HF. (Gaussian 09 and GaussView 5.0.8 [38,39]; Paint/Windows 10; Microsoft Office PowerPoint 2007 software).

Due to the presence of the chiral center in C2, the studied flavanones may exist in the form of two stereoisomers R and S and the geometries of both stereoisomers were optimized with the use of DFT(B3LYP)/6-31G(d,p)/PCM method. Comparing the obtained electronic energy values (see Table S1) it may be noticed that S stereoisomers of the flavanones were about 2 kcal/mol more stable than corresponding the R forms. The DFT(B3LYP)/6-31G(d,p)/PCM optimized geometries (2S-flavanones) are presented in Figure 4 while the selected geometrical parameters of the optimized structures are shown in Table 1.

The discontinuity in the electron conjugation caused by the absence of the C2–C3 double bond in the chromone ring C and the presence of the substituents at C2 (phenyl substituent in case of flavanone, 6HF and 7HF and 2′hydroxyphenyl in the case of 2′HF) leads to the deviation of the part of ring C from planarity. This deviation from planarity can be described by the values of C10C9O1C2 and C9C10C4C3 dihedral angles. In the case of all the studied compounds, with the exception of 2′HF, these values are about −20 and −3 degrees for C10C9O1C2 and C9C10C4C3, respectively (see Table 1). From Figure 4 it may be easily noticed that the structures of the studied flavanones are not planar with the dihedral angles of around −40 and 80 degrees between C and B rings of F, 6 HF and 7 HF. Similar values have been obtained by Shireen et al. [42]. The theoretically predicted geometrical parameters of aqueous 6 HF correlated quite well with the available experimental data [40,41], taking into account that geometry optimization has been performed for a single S stereoisomer in aqueous solution. Examination of the geometry of 6HF reveals easier possibility of interactions of hydrogen bonding leading in aqueous solution to involvement of water molecules in formation of linkage of hydrogen bonds between 6-OH and oxygen carbonyl.

Analysis of the frontier orbitals (HOMO, LUMO) may provide important information for understanding the electronic properties and the reactivity of the molecules. The HOMO and LUMO orbital energies together with the quantum chemical descriptors (determined using orbital vertical method according to the Koopmann’s theorem) are gathered in Table 2. The latter descriptors may be helpful in the assessment of the antioxidant potential of the studied compounds.

**Figure 5:**
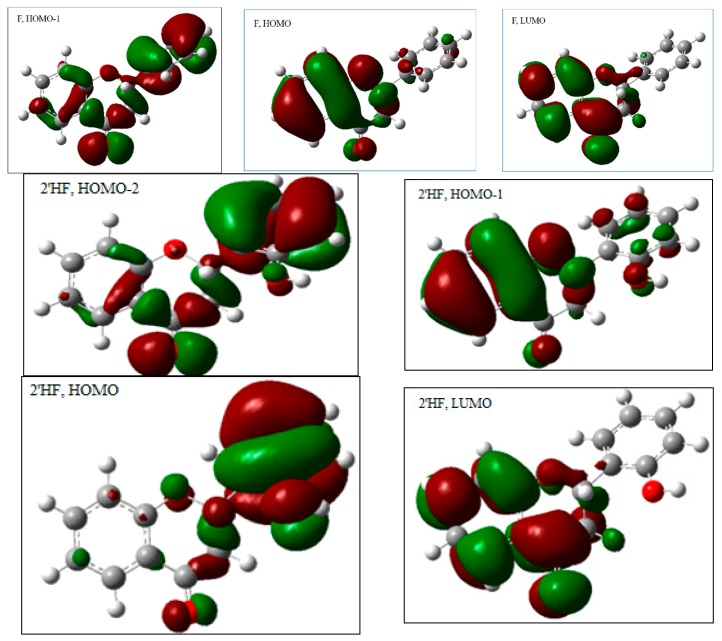

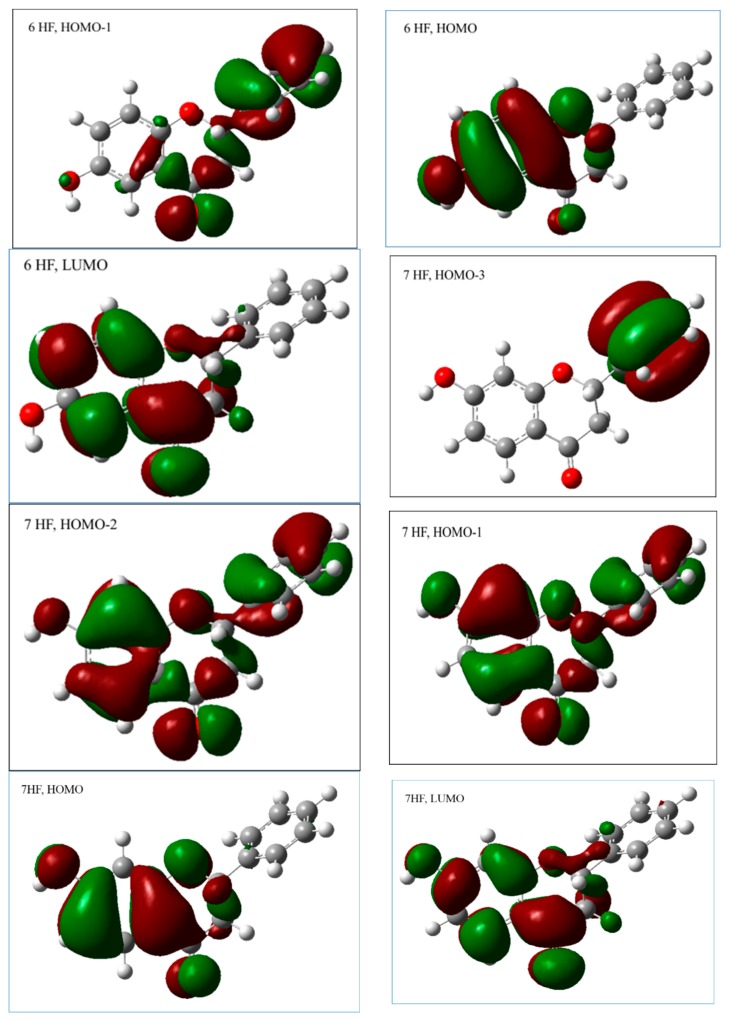
Topologies of molecular orbitals participating in the electronic transitions in the flavanones: F, 2′HF, 6HF and 7HF. (Gaussian 09 and GaussView 5.0.8 Paint/Windows 10; Microsoft Office PowerPoint 2007 software).

The calculated values of chemical quantum descriptors: η, χ, μ, ω and S are similar to those previously calculated for other flavanones [42] and comparable to the values estimated for a well-known anti-oxidant quercetin [43]. The results in Table 2 clearly point out that 6HF can reveal the best antioxidant potentiality with respect to F, 2′HF or 7HF which as our calculation predict can show very similar antioxidant activities. The graphical representations of frontier orbitals are reported in Figure 5.

Taking these facts into account one can conclude that our quantum mechanics calculations support anti-oxidant activity of the studied flavanones as it was found out by experimental data [44,45].

#### 2.2.2. The Theoretically Predicted UV–Vis Characteristic of the Studied Flavanones

**Figure 6:**
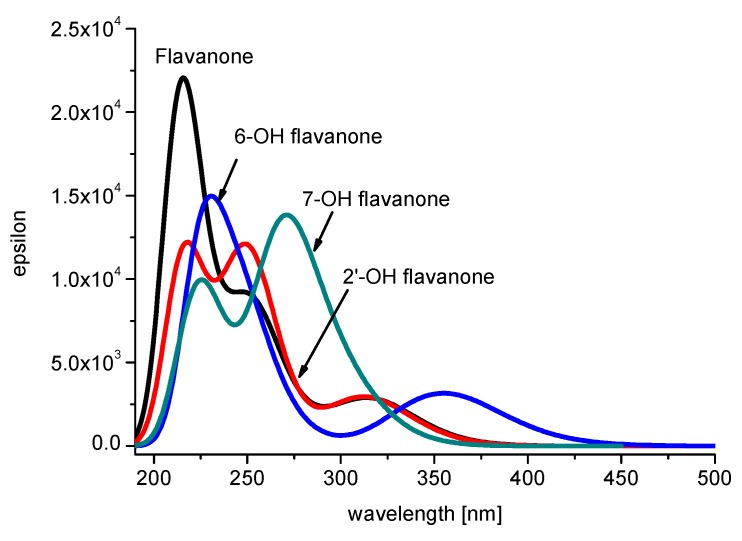
The simulated UV–Vis spectra of the 2S flavanones: flavanone, 6-hydroxyflavanone, 7-hydroxyflavanone and 2′-hydroxyflavanone. (Gaussian 09 and GaussView 5.0.8 Origin(Pro) software).

In polyphenols, π-type molecular orbitals, which are extended over the molecular backbone, are involved in the electronic transitions. Absorption spectra of flavanones usually consist of the broad band (II) located between 270 and 290 nm with a shoulder around 320 nm. Substituents in the flavanone ring have great impact on the location of the absorption maxima [42]. Absorption spectra of F, 2′HF, 6HF and 7HF in water have been calculated within the TD DFT (B3LYP)/6-31+G(d,p)/PCM model using the DFT(B3LYP)/6-31G(d,p)/PCM optimized geometries. The extension of the basis set in the TD DFT calculations is important for better description of the extended π-systems, and the application of 6-31+G(d,p) basis set was the compromise between the time of calculation and the quality of the obtained results. The simulated UV–Vis spectra of the most stable 2S stereoisomers of the studied flavanones are presented in Figure 6, while the spectroscopic parameters of the electronic transitions to the three low-lying singlet excited states are reported in Table 3. 

As expected for enantiomers, the theoretically predicted UV–Vis absorption characteristics of both 2S and 2R stereoisomers of the flavanones are similar. Analyzing the theoretically predicted UV–Vis spectra, it may be noticed that the lowest energy absorption band of 6HF is red shifted with respect to the other compounds. The TD DFT(B3LYP)/6-31+G(d,p)/PCM calculations predicted that the lowest energy absorption band of 6HF arises from single electronic transition of the energy of 3.4669 eV (356.10 nm) and is quite intense (f = 0.0753) as compared to the oscillator strength of the lowest transitions for the other compounds. The energy separation between states S_1_ and S_2_ is higher (0.3 eV) in comparison to the other studied compounds; moreover S_0_→S_2_ has low oscillator strength (f = 0.0049); this transition is probably obscured by the more intense S_0_→S_1_ transition (0.0753). The S_0_→S_1_ transition is the HOMO→LUMO transition of the π→π* character. The topologies of orbitals having considerable contributions in the three low lying electronic transitions in the studied compounds are presented in Figure 5. The HOMO and LUMO orbitals are located mainly on the A and C rings of 6HF, HOMO with high coefficient on the oxygen atom of the 6-OH group. The S_0_→S_2_ and S_0_→S_3_ transitions mainly involve the HOMO-1 and LUMO orbitals. The HOMO-1 orbital is predominantly extended on the B ring and on the carbonyl group of the ring C.

The TD DFT(B3LYP)/6-31+G(d,p)/PCM model predicted that the maximum of the lowest absorption band of the 7HF is around 270 nm (see Figure 6). Analyzing the calculated spectroscopic parameters (Table 3), it can be noticed that 3 transitions contribute to that band: two weak close lying transitions S_0_→S_1_ (f = 0.0044) and S_0_→S_2_ (f = 0.0793) and intense S_0_→S_3_ transition (0.3040) at ~270 nm. The S_0_→S_1_ transition is the mixture of transitions: HOMO-3→LUMO; HOMO-2→LUMO and HOMO-1→LUMO and HOMO→LUMO. The calculations predicted that S_1_ and S_2_ states are very close in energy (the energy separation between them is about 0.2 eV); the TD DFT (B3LYP) level of theory has some limitations in the description of the photophysics of such close-energy states; that is the TD DFT method overestimates the excitation energies of such transitions, especially for chromophores with extended π-electrons [26]. The S_0_→S_2_ transition of 7HF is mainly HOMO→LUMO transition, while S_0_→S_3_ transition primarily involves transition between HOMO-1 and LUMO. The HOMO and LUMO orbitals are predominantly localized on the ring A and C with considerable contribution on the oxygen atom of the 7 OH group.

The calculated long-wavelength absorption band of flavanone F consists of two close lying (0.16 eV) transitions S_0_→S_1_ and S_0_→S_2_, wherein the S_0_→S_1_ transition is very weak (f = 0.0047) and obscured by the intense S_0_→S_2_ transition (f = 0.0656). The S_0_→S_1_ and S_0_→S_3_ electronic transitions are mainly HOMO-1→LUMO transitions, while S_0_→S_2_ is predominantly HOMO→LUMO transition. The LUMO orbital of F is located on the A and C rings; the HOMO orbital also but additionally with small coefficients on the ring B. The HOMO-1 orbital of F has large contributions on the B ring and also extends. It is delocalized over the A and C rings.

**Figure 7:**
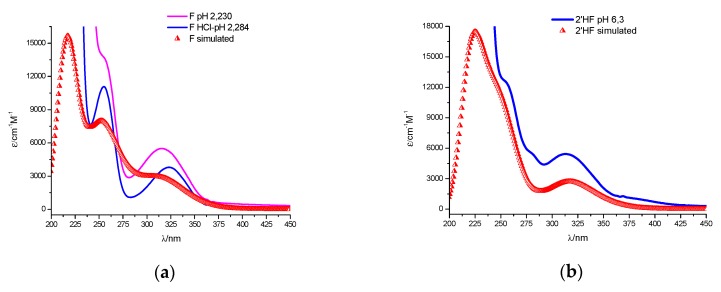

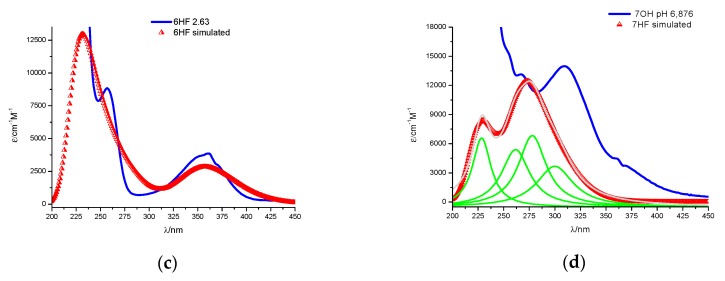
Experimental and simulated UV–Vis spectra of the flavanones: (**a**) F, (**b**) 2′HF, (**c**) 6HF and (**d**) 7HF. (Origin(Pro) software using the original files from the instrument: Perkin-Elmer Lambda 11 spectrophotometer).

In the case of the 2′HF the lowest energy absorption band arises from three low lying (~0.1 eV) transitions S_0_→S_1_, S_0_→S_2_ and S_0_→S_3_. The S_0_→S_1_ is less intense as compared to the S_0_→S_2_. The S_0_→S_1_ transition is the HOMO→LUMO transition, while S_0_→S_2_ and S_0_→S_3_ are the HOMO-1→LUMO and HOMO-2→LUMO, respectively. The HOMO-1 and LUMO orbitals of 2′HF are localized mainly on the A and C rings; LUMO with smaller coefficient on ring B with respect to HOMO-1. The HOMO orbital is mainly localized on the ring B; while the HOMO-2 on the B and C rings. The theoretically predicted short wavelength absorption band of 2′HF arises from two electronic transitions: the less intense (f = 0.0072) HOMO-2→LUMO transition of the energy of 4.6318 (267.68 nm) most probably is suppressed by the intense transition of predominant HOMO-3→LUMO (f = 0.1920) located at 251.89 nm. Comparing the theoretically predicted and experimental spectra of 7HF at pH 6.8 it can be observed that the TD DFT(B3LYP)/6-31G+(d,p) calculated excitation energies are overestimated which is a typical tendency error of the applied theoretical model (Figure 7).

The experimental and simulated spectra of F, 2′HF and 6HF are similar to each other (Figure 7). A pronounced difference occurs in the spectrum of 7HF. It consists of a long-wavelength band with maximum at around 310 nm and a shoulder at around 375 nm on the red region of the spectrum. It may arise from two theoretically predicted transitions S_0_→S_3_ (~270 nm) and S_0_→S_2_ (~300 nm). The shoulder of the long-wavelength of the experimental spectrum at pH 6.8 may be attributed to the theoretically predicted S_0_→S_1_ transition (318 nm), which is characterized by the very low oscillator strength and it may be suppressed by the more intense S_0_→S_2_ transition. It can be noticed from the deconvolution of the theoretical spectrum of 7HF that the three lowest energy gauss-shape bands (at around 300, 275, 265 nm) could be attributed to the theoretically predicted S_0_-S_1_, S_0_-S_2_ and S_0_-S_3_ transitions at 317, 300 and 270 nm, respectively. The higher energy shoulder arises from the theoretically anticipated electronic transition at 221.73 nm (Figure 7).

UV–Vis spectra together with theoretical calculations give information about structural changes in molecules, which are very important in explanation of biological activity of the compounds.

#### 2.2.3. The Electronic Structures of the Chalcones Predicted by the DFT(B3LYP)/6-31G(d,p)/PCM Method

**Figure 8:**
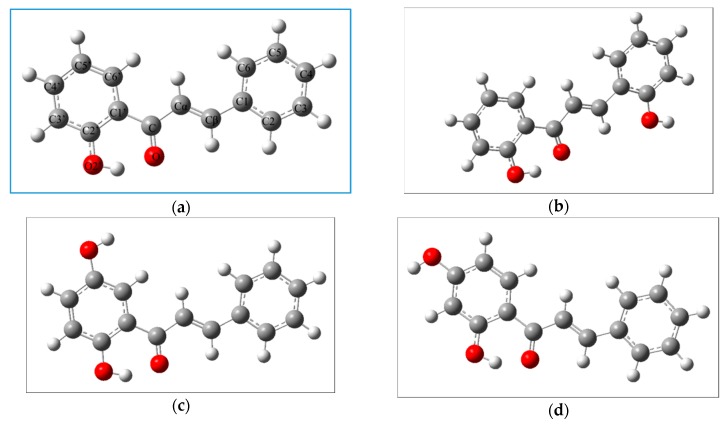
The DFT(B3LYP)/6-31G(d,p)/PCM optimized geometries of the chalcones: (**a**) 2′-OH chalcone (derived from F); (**b**) 2-OH chalcone (derived from 2′HF); (**c**) 5′-OH chalcone (derived from 6HF); (**d**) 4′-OH chalcone (derived from 7HF). The atom numbering is presented for 2′-OH chalcone (derived from F); the same is for 2-OH chalcone, 5′-OH chalcone and 4′-OH chalcone. (Gaussian 09 and GaussView 5.0.8 Paint/Windows 10; Microsoft Office PowerPoint 2007 software).

In order to provide more insight into the UV–Vis spectra of the studied flavanones in basic solutions, the structures of corresponding chalcones have been optimized with the DFT(B3LYP)/6-31G(d,p)/PCM model and the most stable structures are presented in Figure 8, while the selected geometrical parameters of the optimized structures are reported in Table 4. The α,β-double bond is in the trans configuration due to the strong steric effect between the B-ring and the carbonyl group in the most stable configuration of chalcones. Analyzing the optimized structures it is evident that the geometries of the studied chalcones are nearly planar (Table 4) as it has been previously determined by Serdiuk et al. [16]. The calculated geometrical parameters suggest the possibility of the formation of intramolecular hydrogen bond between hydroxy group O2′-H and carbonyl oxygen, which favors planar geometry and may facilitate the occurrence of intramolecular proton transfer processes upon excitation [16].

**Figure 9:**
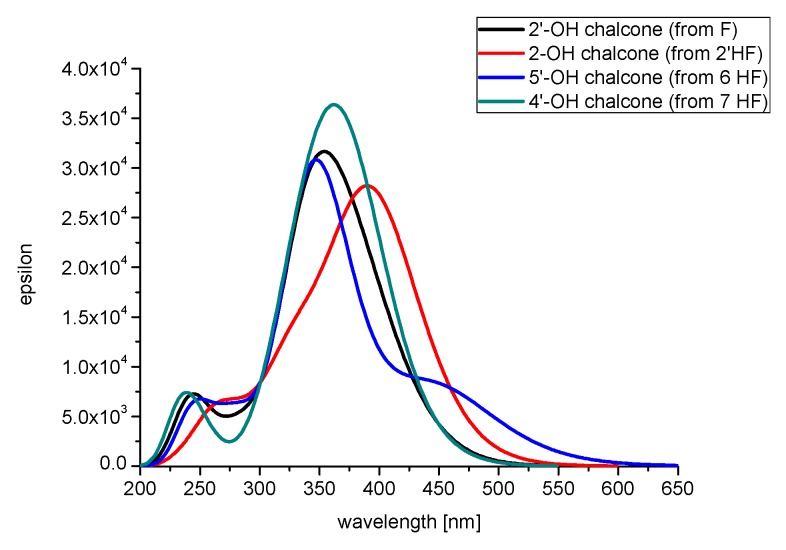
The simulated UV–Vis spectra of the chalcones: 2 OH chalcone (2′HF), 5′ OH chalcone (6HF), 4′ OH chalcone (7HF) and 2′ OH chalcone (F). (Gaussian 09 and GaussView 5.0.8; Origin(Pro) software).

Due to the importance of π electron delocalization in chalcones, the TD DFT calculations have been performed applying extended 6-31+G(d,p) basis set and with the use of the DFT(B3LYP)/6-31G(d,p) geometries. The simulated UV–Vis spectra of the studied chalcones are presented in Figure 9, while the spectroscopic parameters of the electronic transitions to the three low-lying singlet excited states are reported in Table S2.

In the case of the chalcone formed from 7HF the results of calculations showed quite good agreement of the lowest energy absorption band with the experimental data: the long wavelength absorption band of the chalcone formed from 7HF with maximum at 358 nm corresponds to the computationally predicted maximum at around 360 nm (Figure 9). The applied theoretical model overestimated the S_0_→S_1_ excitation energies for chalcones derived from F and 2′HF. The experimentally obtained maxima of the long wavelength absorption band of F and 2′HF at pH 11 are located at 410 and 425 nm, respectively, whereas the TD DFT model predicted that the maxima of the long wavelength absorption are located at around 355 and 390 nm for chalcones of F and 2′HF, respectively (Figure 9). This discrepancy between theoretical and experimental data may be rationalized by the limitation of the applied B3LYP functional in the theoretical description of molecular system with extended π electron delocalization [26]. Moreover, in the applied TD DFT model only electronic transitions to the excited state Franck-Condon states (without any changes in the ground state geometry upon excitation) are considered. The ground-state geometries of 2′hydroxychalcones are planar and this planarity is related with the formation of the intramolecular hydrogen bond between O2′ –H and carbonyl oxygen. This hydrogen bond is weakened upon excitation and the molecule geometry becomes twisted [16]. There is a possibility of the occurrence of intramolecular proton transfer processes leading to formation of phototautomers. The theoretically predicted long-wavelength band of 2′OH chalcone arises from S_0_→S_1_ and S_0_→S_2_ electronic transitions located at (3.211 eV) 386.13 and (3.604 eV) 343. 99 nm. These excitation energies are underestimated as compared to the previously [16] calculated (using TD DFT(M062X) method) values for this compound (3.32 eV (373 nm) and 3.36 eV (369 nm) for S_0_→S_1_ and S_0_→S_2_ electronic transitions, respectively).

In the case of chalcone of 6HF, the theoretically predicted S_0_→S_1_ transition located at 447 nm may be attributed to the experimental long-wavelength maximum of 6HF in solutions of pH 11 located at 398 nm; while the experimental short-wavelength band with the maximum at 304 nm may correspond to the theoretically predicted S_0_→S_3_ transition located at 311 nm. The applied theoretical model generated lower excitation energies and caused underestimation. It may be explained by the fact that no specific hydrogen bonding solute–solvent interactions (which are of a great importance for 6HF) were taken into account within the applied theoretical model.

**Figure 10:**
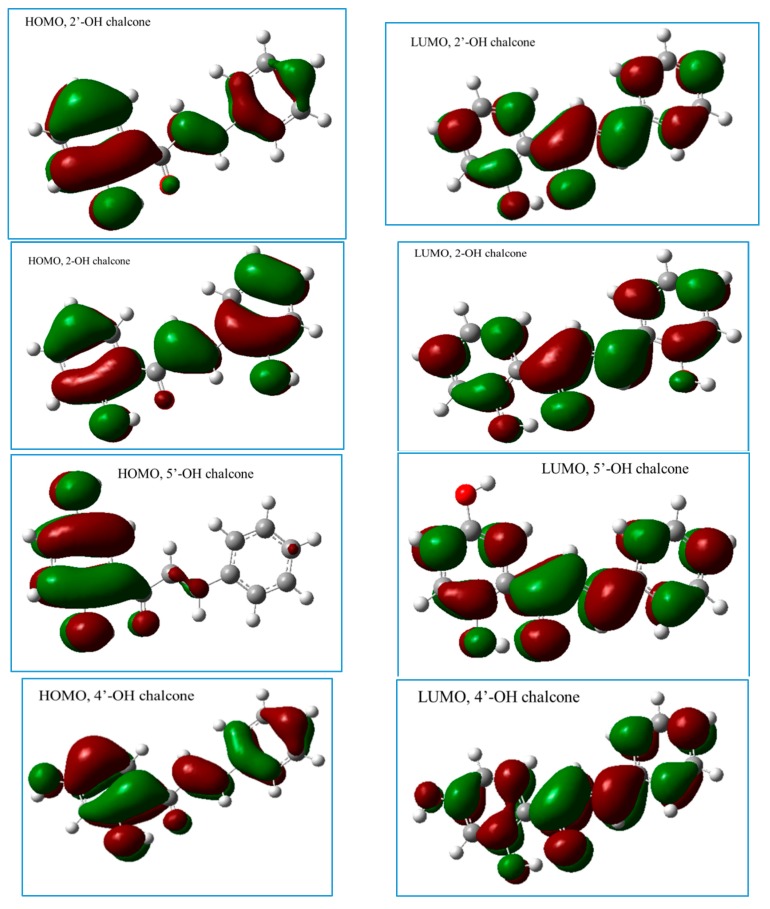
The graphical representation of frontier orbitals of the chalcones derived from F, 2′HF, 6HF and 7HF. (Gaussian 09 and GaussView 5.0.8; Paint/Windows 10; Microsoft Office PowerPoint 2007 software).

The calculations showed that the long wavelength S_0_→S_1_ transition of the chalcones under study is a HOMO→LUMO transition, while the S_0_→S_2_ transition is predominantly the HOMO-1→LUMO. The graphical representation of the frontier orbitals (HOMO, LUMO) of the studied chalcones is included in Figure 10.

It can be seen that the frontier orbitals of the chalcones under study with the exception of the HOMO orbital of 5′-OH chalcone (derived from 6HF) are mainly delocalized on the whole molecule. Comparing the calculated values of the quantum chemical descriptors (IP values, HOMO-LUMO energy gap; see Table S3) it may be suggested that the chalcone derived from 6HF should exhibit better antioxidant activity. Based on the determined IP values the predicted antioxidant activity order might be represented as follows: 5′OH chalcone (from 6HF)>2-OH chalcone (from 2′HF)>4′OH chalcone (from 7 HF)>2′OH chalcone (from F).

### 2.3. Cyclic Voltammetry

Cyclic voltammetry responses were recorded at a Pt disk electrode at a potential scan rate of 100 mV/s. An example of a voltamogram is given in Figure S2. All responses show broad and poorly reproducible oxidation processes in the range 0.7–1.1 V, with an associated reduction process between 0.2 and −0.3 V. Experiment performed at potential scan rates between 10 and 100 mV/s indicates an increase of the anodic peak current and a shift towards more positive potential values, implying a diffusion controlled and a no-reversible electron transfer process, probably followed by a chemical reaction (Figure S2). Such a result substantiates the specific structure of the flavanones here investigated, containing only a hydroxyl substituent in different positions of rings A or B. In fact, the redox behavior of flavanone’s analogues, as well as the corresponding flavonols (containing a double bond between atoms C2 and C3), indicates usually a two-electron process associable to the presence of hydroxyl groups in orto- or para-positions on the aromatic rings A or B, mainly responsible of their antioxidant activity [46]. According to computational results here reported, as well as literature data on analogues of flavones [47] we could reasonably predict a higher antioxidant activity of 6HF than 7HF, 2′HF and non-substituted flavanone F.

### 2.4. Acid–Base Profiles of Flavanones Derived from Potentiometric Titration

The acid dissociation constant (pK_a_) is one of the most commonly used physicochemical parameters, and its determination is the subject of interest in a wide range of scientific areas. The dissociation constant is of broad importance in biological systems, in synthetic chemistry, and pharmacology [48]. In this study, the pK_a_ values of three flavanones 6HF, 7HF and 2′HF have been determined in methanol–water binary mixture (40%/60% *v*/*v*) by potentiometric methodology. Measurable quantities of isomers i.e., flavanone/its hydroxyl-derivative—chalcone have been found in solutions containing 6HF, 7HF, 2′HF within a pH range of 10.5 to 13.

A Binary solvent such as a methanol/water mixture was chosen due to its lower polarity than pure water, while having a similar environment. The pK_a_ value at one unique methanol/water mixture is recommended because it is faster and simpler with respect to others for example to the classical Yasuda–Shedlovsky plot [49]. Therefore, a mixed solvent with a ratio of 40%:60% v/v of methanol/water was used in these studies. Such a ratio is recommended by some authors as the least error-prone of mixed solvents because of a greater accumulation of information about the behavior of the glass electrode in this solvent [50]. The pK_a_ values of 6HF, 7HF, 2′HF obtained in this medium have been compared with those given in the literature, and also with the values predicted by the DFT calculations. Potentiometric methodology has allowed estimating proton ionization constants of hydroxychalcones although the values determined should be considered carefully due to the electrode error in very high pH range. The results of the determined pK_a_ are summarized in Table 5 (examples of titration curves are attached in Supplementary Data, Figure S3).

**Figure 11:**
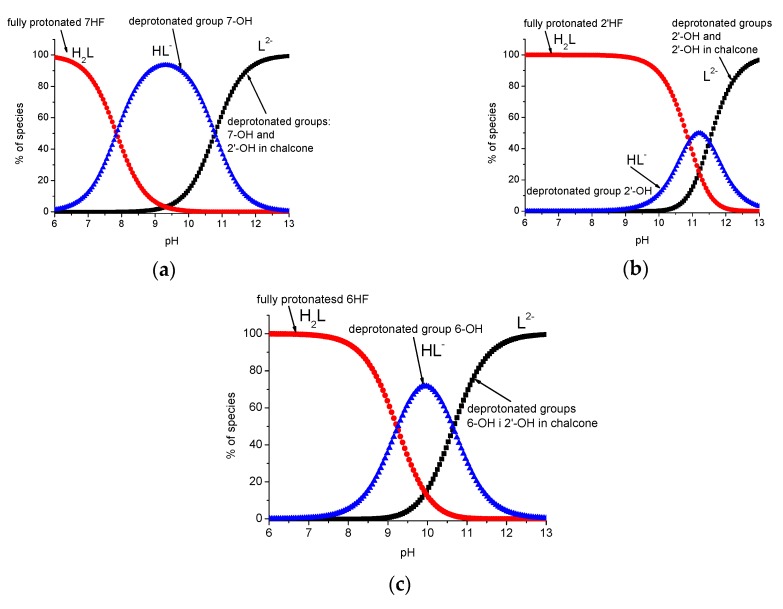
Species distribution diagram showing the species of (**a**) 7HF, (**b**) 2′HF and (**c**) 6HF formed as a function of pH. All reactants are 1 × 10^−3^ M in concentration.

The flavanones show different pK_a_ values. The determined pK_a_ values result from different molecular geometries and differences in charge density distributed in the rings. The deprotonation of 6-OH group is more difficult in 6HF due to possible interaction with water molecules through formation of hydrogen bonds. Examination of the geometry of 6HF revealed an impact of water molecules in formation of linkage of hydrogen bonds between 6-OH and oxygen carbonyl in aqueous solution. The formation of the bonds hinders the deprotonation of the 6-OH group. Therefore, this group releases a proton at higher pH and the resulting pK_a_ value is higher. On the other hand, in the case of 7HF the molecular structure (lack of CO group in proximity) promotes deprotonation of the 7-OH group at lower pH and, therefore, the pK_a_ value of this group is much lower compared to other monohydroxy flavanones. In the case of 2′HF, the hydroxy group is localized in aromatic B ring which is unconjugated with A and C rings due to lack a double bond between C2 and C3. It leads to charge localization in this ring and the consequence is stronger attraction of hydrogen in 2′-OH group. Therefore, the group releases proton at high pH region with relatively high pK_a_ value. Representations of protonated/deprotonated forms of 6HF, 7HF, 2′HF are shown in species distribution diagrams (Figure 11). The presence of species was found according to methodology described elsewhere [52].

One can see from the diagrams that the protonated and partially deprotonated (in ca. 20%) forms of 7HF and the fully protonated forms of 6HF and 2′HF dominate at neutral pH region. Domination of mono-deprotonated HL-form of all flavanones prevails in basic region. Chalconate anions can be present above pH 12. It is clearly seen comparing species distribution diagrams that different forms of the 6HF, 7HF and 2′HF (protonated or deorotonated) can participate in biological action at physiological pH. Hence one can predict bioavailable forms of the compounds in physiological fluids. The pK_a_ values obtained in this work that are attributed to 6HF, 7HF, 2′HF or their corresponding chalcones show differences with comparison to those reported in literature. This is due to the fact that various applied methodologies of measurements and calculations usually provide different results. However, it should be considered that potentiometry gives good accuracy and precision because the electrode system is calibrated before each potentiometric titration and the electrode remains into the solution during all the titration, so that the calibration parameters are the same for all titration data corresponding to the same experimental run [48].

The theoretically predicted pK_a_ values for 7 HF and 2′HF are in a quite good agreement with those resulting from experiments. Surprisingly, the applied theoretical model considerably overestimated the pK_a_ value for 6HF. The same tendency was observed in the theoretically predicted proton affinities calculated from the DFT(B3LYP)/6-31G(d,p)/PCM enthalpies for neutral and anionic form of flavanones: 58.826, 61.400 and 52.989 kcal/mol for 2′HF, 6HF and 7HF, respectively (enthalpy of proton taken from [53], solvation enthalpy of proton in water taken from [54]). This theoretical prediction is contradictory to both the experimental results and the literature data which clearly showed that the proton of 6-OH group has more acidic character. Since no specific solute-solvent interactions (which seem to be crucial in the acid–base equilibria of 6HF) are taken under consideration of the applied theoretical model, the calculated pK_a_ value for this flavanone is substantially different in comparison to the others. Moreover, the pK_a_ value of an acid group in a molecule could be estimated as the sum of two terms, the first corresponding to the pK_a_ of the acid group or reaction center, and the second indicates the change in the ionization behavior as consequence of a perturber structure, meaning any molecular structure appended to acid group. The second term is factored into mechanistic components, describing the differential resonance, electrostatic, solvation and hydrogen bonding of perturber structure with the protonated and unprotonated states of acidic group, respectively. In our model, not all factors were included, which in turn could have led to an overestimated value compared to that of experimental one.

### 2.5. Absorption Spectroscopic Measurements of DNA

The interactions between small molecules and DNA are commonly investigated by electronic absorption spectroscopy since binding to the macromolecule leads to changes in the electronic spectrum [55]. The interaction of F, 2′HF, 6HF and 7HF with CT DNA was studied keeping the concentration of respective flavanone fixed and adding DNA in aliquots of required amount up to a concentration of 3.32 × 10^−5^ M. An increase in absorbance has been observed for F, 2′HF and 6HF. In the case of 7HF hypochromism is clearly seen in the spectra (Table 6). The values of the K_b_ intrinsic constants determined from the Wolfe–Shimer diagrams are in Table 6.

The appearance of isosbestic point in the spectrum of 7HF may indicate that: (i) the compound binds to DNA in a single mode, (ii) the presence of a new species formed during the interaction, (iii) it enables the assumption of two-state system consisting of bound and free 7-hydroxyflavanone species in the binding process that are in equilibrium [56,57].

The both effects hyperchromism and hypochromism occur without shifts of λ_max_. Such spectral profiles indicate weak interactions with CT DNA [58]. This is also confirmed by the values of K_b_ intrinsic binding constants of the flavanones (Table 6). Slight differences between them point out a similar mode of interaction which may be electrostatic or hydrogen bonding [59]. The values of the K_b_ intrinsic constants are in the following order 7HF (4.81 × 10^4^ M^−1^)>2′HF (4.65 × 10^4^ M^−1^)>F (4.58 × 10^4^ M^−1^)>6HF (4 × 10^4^ M^−1^) and demonstrate weak interaction with CT DNA. They are close to each other, although in the case of 7HF the observed changes in the spectrum may signify a greater affinity for DNA compared to the others. Weak interactions with CT DNA indicate that the anti-tumor activity of these flavanones is unlikely to occur by destroying the structure of DNA, but rather through their interaction with cancer cell components such as enzymes. Enzyme inactivation revealed in the cytotoxic activity of 6HF and 2′HF [reported in [9,10] seems to confirm this conclusion.

## 3. Materials and Methods

### 3.1. Reagents

The racemic flavanone, 2′-, 6-, 7-hydroxyflavanones, NaOH, NaCl, KNO_3_, HNO_3_, HCl methanol (CH_3_OH) and all other compounds were purchased from Sigma-Aldrich Co. (Poznań, Poland). All reagents were of analytical quality and were used without further purification.

### 3.2. Electrochemical Measurements

#### 3.2.1. Potentiometry

There are several methods for the determination of dissociation constants. Traditionally, potentiometry has been the most useful technique for the determination of equilibrium constants [60,61] because of its accuracy and reproducibility. Moreover, the use of computer programs for the refinement of equilibrium constants allows the different pK_a_ values of polyprotic substances to be determined, even when they are very close [62]. In order to overcome the lack of information related with the acid–base equilibria of monohydrated flavanones, the pK_a_ values of 6-hydroxy-, 7-hydroxy- and 2′-hydroxyflavanones have been determined by means of potentiometric measurements in methanol–water binary mixture. To determine pK_a_ values, emf (*electromotive force*) was measured with a precision of ±0.1 mV, using an automated system Molspin pHmeter (Molspin Ltd., Newcastle-upon-Tyne, UK) equipped with a digitally operated syringe (the Molspin DSI 0.250 mL) controlled by a PC computer, using a Russel CMAWL/S7 semi-micro combined electrode. The titrations were done with carbonate-free NaOH solution of accurately known concentration (ca. 0.1 M). The concentrations of the base and HCl or HNO_3_ solutions were determined by pH-potentiometric titrations. The electrode system was calibrated according to Irving et al. [63]. The pH-metric readings could, therefore, be converted into hydrogen-ion concentrations. The average water-ionization constant, pKw was 13.78 ± 0.05 with methanol/water (40%/60%, *v*/*v*) as solvent [64]. This value is similar to that presented in the literature (pK_w_ = 13.71) [65]. A slight difference results from the use of different measurement conditions as well as the assumptions made in the calculation. The samples were deoxygenated by bubbling purified argon for ca. 10 min prior to the measurements, as well as during the titrations. The pH-metric titrations were carried out in the pH range 2.0–12.0 and the initial volume of the samples was 2.0 mL. The flavanones’ concentration was 1 × 10^−3^ M. The flavanones’ stock solutions were determined by the Gran’s method [66]. The accepted fitting of the titration curves was always less than 0.01 mL. The number of experimental points was within 100–150 for each titration curve. The reproducibility of the titration points included in the evaluation was within 0.005 pH units in the whole pH range examined. Dissociation constants of hydroxyl groups of flavanones were evaluated by iterative non-linear least squares fit of the potentiometric equilibrium curves through mass balance equations for all the components, expressed in terms of known and unknown equilibrium constants using the computer program SUPERQUAD [67]. The value obtained for sigma (the root mean squared weighted residual), after refinement of the stability constants, was ≤1, which means that the data was fitted within experimental error. The equilibrium constants reported in this work were obtained from fittings that used three titration curves simultaneously (examples of titration curves are included in Figure S1.

#### 3.2.2. Cyclic Voltammetry

Cyclic voltammetry tests were performed in a three-electrode single-compartment cell. Working, counter and reference electrodes were a Pt disk (diameter 2 mm), a Pt gauze, and aqueous Ag/AgCl (KCl 3M), respectively. A proper amount of a MeOH solution of each flavanone was added in 5 mL of an aqueous saline phosphate buffer solution pH 7.4 (a 1 L aqueous solution contains NaCl 8.00 g, KCl 0.20 g, Na_2_HPO_4_·2 H_2_O 1.44 g, KH_2_PO_4_ 0.24 g). Oxygen was removed by flowing Ar through the solution before each experiment, and Ar flow was kept over solutions during the voltammetric tests. 

### 3.3. UV–Vis Spectrophotometric Experiments

UV–Vis spectrophotometric pH titrations were carried out with solutions containing 2 × 10^−5^ M of flavanone, 6-hydroxyflavanone, 7-hydroxyflavone and 2′-hydroxyflavanone), 6.25 × 10^−3^ M HNO_3_ or HCl, 0.1 M NaCl and 0.5% of DMSO by using a Perkin-Elmer Lambda 11 spectrophotometer in the λ interval 200–900 nm. Solutions were inserted in a quartz cell with a path length of 1 cm. The same apparatus was used in DNA experiments.

Deoxyribonucleic acid sodium salt from Calf Thymus DNA (CT DNA) was purchased from Sigma (#D3664). The concentration of CT DNA was determined from the absorption intensity at 260 nm with ε value of 6600 M^−1^ cm^−1^. A Tris buffer (5 mM Tris-HCl, 50 mM NaCl, pH 7.2) was used and UV–Vis spectra were recorded after each addition of concentrated CT DNA stock to 25 µM solutions of flavanones in a quartz cuvette (path length = 1 cm), at 25 °C. The binding ability of molecules with CT DNA can be estimated through the K_b_ intrinsic binding constant, which was obtained by monitoring the λ_max_ with increasing concentrations of CT DNA. The K_b_ values are given by the ratio of slope to the y intercept in plots [DNA]/(εA − εf) versus [DNA], according to the Wolfe–Shimer equation:

[DNA]/(ε_A_ − ε_f_) = [DNA]/(ε_b_ − ε_f_) + 1/(K_b_(ε_b_ − ε_f_)) (1)

where [DNA] is the concentration of DNA in base pairs, ε_A_ = A_obsd_/[compound], ε_f_ = the extinction coefficient for the free compound and ε_b_ = the extinction coefficient for the compound in the fully bound form [68].

### 3.4. Computational Methods and Softwares

All calculations were performed using the Gaussian 09 [38] and GaussView 05 software package [39]. Geometry optimization of the studied compounds has been performed with the use of DFT applying the hybrid B3LYP functional [20,21,22] and the 6-31G(d,p) basis set. Solvent effects (water) have been included in the optimization calculations within the framework of Polarizable Solvation Model (PCM). Vibrational frequency calculations (at the same level of theory) have been carried out (optimization with the opt+freq DFT(B3LYP)/6-31G(d,p)/ PCM (water) keyword with the default Berny algorithm [69]). No imaginary vibrational frequency has been found which indicated that the optimized geometries corresponded to the local minima on the potential energy hypersurface.

The ionization potential IP and electron affinity EA values were calculated by orbital energy method (according to Koopmann’s theorem): IP = −E_HOMO_, EA = −E_LUMO_. Energies of the HOMO and LUMO orbitals were calculated using DFT(B3LYP)/6-31G(d,p)/PCM (water) method. Ionization potential IP is defined as the energy which is required to remove an electron, while electron affinity EA is the energy released when the electron is added. Having the IP and EA, the quantum chemical descriptors (electronegativity χ, hardness η, softness S, chemical potential μ, and electrophilicity index ω) were calculated according to the following formulas:

(2) $$\chi =\frac{IP+EA}{2}$$

(3) $$\;\omega =\frac{\mu^{2}}{2\eta }\;$$

(4) $$\;S=\frac{1}{2\eta }\;$$

(5) $$\;\eta =\frac{IP-EA}{2}\;$$

(6)  μ=−χ 

Hardness η describes the resistance to charge transfer, while the inverse of hardness is given by softness S [70]. Electronegativity χ is the measure of the tendency to attract bonded electron pairs; while the electrophilicity index ω describes the affinity of electrons [71]. Chemical potential is the measure of tendency of an electron to escape from equilibrium [72].

The UV–Vis spectra and the spectroscopic parameters of the electronic transitions to the lowest excited singlet states have been obtained from the TD (nstates = 10) DFT(B3LYP)/6-31+G(d,p)/PCM(water) calculations using the DFT(B3LYP)/6-31G(d,p)/PCM(water) optimized geometries. The TD DFT (B3LYP)/6-31+G(d,p) calculations of the vertical excitations have been performed using the ground state equilibrium geometries with the linear response, non-equilibrium solvation. The reported results are for vertical transitions thus no change in geometry between the ground and excited states has been included.

The pK_a_ values were calculated based on the free Gibbs energy of the following reaction: HF_(s)_→A_(s)_**^−^** + H_(s)_**^+^** according to the well-known formula:

(7) $$pK_{a}=\frac{\Delta G_{a}^{*}}{RTln\left(10\right)}$$

where

(8) $$\Delta G_{a}^{*}=G^{*}\left(A_{\left(s\right)}^{-}\right)+G^{*}\left(H_{\left(s\right)}^{+}\right)-G^{*}\left(HF_{\left(s\right)}^{}\right)\;\;\;$$

and the subscript “(s)” indicates solvated species. Our preliminary calculations showed that extension of basis set from 6-31G(d,p) to 6-31+G(d,p) had minor impact on the obtained geometries, however, the use of 6-31+G(d,p) basis set gave better results in determining the pK_a_ values. The total free Gibbs energies of the neutral $\left(G^{*}(HF_{\left(s\right)}^{}\right)$ and anionic $G^{*}\left(A_{\left(s\right)}^{-}\right)$ forms of the studied flavanones at standard conditions were calculated as the sum of the DFT(B3LYP)/6-31+G(d,p) calculated thermal correction to free Gibbs energy (in the gas phase) and the DFT(B3LYP)/6-31+G(d,p)/PCM single point electronic energy, similarly to the method previously proposed by Wright et al. [73] and Bryantsev et al. [74]. Proton free enthalpy (energy) in water was calculated using the value −26.28 kJ/mol for proton free energy in the gas phase [53] and the value of −267.9 kcal/mol for proton free energy of solvation previously proposed by Bryantsev et al. [74]. The same procedure has been applied for the determination of the pK_a_ values for the chalcones under study.

The following software packages were used in this work: ChemDraw Version 15.0.0.106 PerkinElmer Informatics [75]; Origin(Pro), Version 2007. OriginLab Corporation, Northampton, MA, USA [76].

## 4. Conclusions

The results of our research have shed some light on clarification of different biological activity of the flavanones studied. As the spectral results both experimental and theoretical indicate the compounds have revealed different susceptibility to microenvironment, i.e., to components of the solution or changes in the concentration of hydrogen ions whose presence is of crucial importance in cellular compartments. This is especially seen in spectral profiles of 6HF (Figure S2). This finding correlates with its stronger cytotoxic effect compared to the other studied flavanones. It leads to the conclusion that this compound can react with cell components, such as enzymes or apoptosis proteins.

Based on the calculated HOMO and LUMO energies, together with cyclic voltammetry data, it was possible to make predictions of reactivity of 6HF, 2′HF 7HF and F. Analyzing the calculated quantum chemical descriptors, it can be suggested that based on the lowest HOMO-LUMO gap and the lowest IP, 6-hydroxyflavanone is expected to be the best antioxidant in this set of monohydroxy-flavanones. Structure–activity relationships observed for antioxidant activity and DNA interactions suggest that 6-hydroxyflavanone can protect DNA against oxidative damage more effectively than flavanone, 2′-hydroxyflavanone or 7-hydroxyflavanone.

Weak interactions with CT DNA indicate that the anti-tumor activity of these flavanones is unlikely to occur by destroying the structure of DNA, but rather through their interaction, e.g., with apoptotic proteins in cancer cells.

To summarize, we have shown that even such subtle changes as altering the location of the OH group from the C6 position to the C7 position in 6HF and 7HF, respectively, lead to significant changes in the spectral profile, which means changes in the electronic structure of the flavanone rings, and this is reflected in their biological activity.

## Figures and Tables

**Table 1:** The selected geometrical parameters of the DFT(B3LYP)/6-31G(d,p)/PCM optimized structures. The atom numbering is the same as for F in Figure 4.

Compound	C10C9O1C2 [°]	C9C10C4C3 [°]	O1C2C1′C6′ [°]	C3C2C1′C6′ [°]
F	−21.055	−2.945	−43.348	78.770
2′HF	−14.690	−7.783	−23.689	98.713
6HF	−22.449	−2.431	−43.553	78.464
6HFexp ^1^	−18.892	−4.058	−55.323	77.974
7HF	−21.531	−2.313	−44.582	77.547

^1^ Crystallographic data taken from The Cambridge Crystallographic Data Centre (CCDC) [40]; deposition number 1225194, [41].

**Table 2:** Quantum chemical descriptors for F, 2′HF, 6HF and 7HF (hardness (η); electronegativity (χ); chemical potential (μ); electrophilicity index (ω); softness (S)) calculated from ionization potential (IP) and electron affinity values (EA), which were estimated by orbital vertical method.

Compound	E_HOMO_^2^ [eV]	E_LUMO_^2^ [eV]	HOMO-LUMO gap [eV]	IP [eV]	EA [eV]	η	χ	μ	ω	S
F	−6.40	−1.70	4.70	6.40	1.70	2.35	4.05	−4.05	4.05	0.21
2′HF	−6.15	−1.67	4.48	6.15	1.67	2.24	3.91	−3.91	3.91	0.22
6HF	−5.87	−1.74	4.13	5.87	1.74	2.07	3.81	−3.81	3.81	0.24
7 HF	−6.32	−1.48	4.84	6.32	1.48	2.42	4.05	−4.05	4.05	0.21

^2^ calculated at the DFT(B3LYP)/6-31G(d,p)/PCM(water) method level of theory.

**Table 3:** The TD (nstates = 10) DFT(B3LYP)/6-31+G(d,p)/PCM calculated spectroscopic parameters (transition electric dipole moment (μ); wavelength corresponding to the excitation energy (λ) and oscillator strength (f)) of the electronic transitions to the three low-lying excited singlet states of 2S stereoisomers of F, 2′HF, 6HF and 7HF.

2S Stereoisomer	S_0_→S_1_			S_0_→S_2_			S_0_→S_3_		
	μ [D]	λ [nm]	f	μ [D]	λ [nm]	f	μ [D]	λ [nm]	f
F	0.0510	327.98	0.0047	0.6799	314.66	0.0656	0.1213	271.61	0.0136
2′HF	0.0292	333.84	0.0027	0.5159	313.93	0.0499	0.2120	304.44	0.0208
6HF	0.8831	356.10	0.0753	0.0528	327.79	0.0049	0.0794	275.33	0.0088
7HF	0.0463	317.88	0.0044	0.7863	300.10	0.0793	2.7015	269.90	0.3040

**Table 4:** The selected geometrical parameters of the most stable structures of the studied chalcones. The atom numbering is the same as for 2′-OH chalcone in Figure 8a.

Compound	C2′C1′Cα [°]	CαCβC1C2 [°]	O2′…O [Å]	O2′H…O [°]
2′ OH chalcone (from F)	180.00	−179.99	2.52	150.81
2′ OH chalcone (from F)	179.90	−177.15	2.52	146.53
exp. Data ^3^	173.48	−174.67	2.53	149.76
2-OH chalcone (from 2′HF)	−179.82	−179.02	2.51	151.15
5′ OH chalcone (from 6HF)	−179.99	−179.98	2.52	150.51
4′ OH chalcone (from 7HF)	180.0	−179.97	2.51	151.74

^3^ Geometrical parameters taken from crystallographic structures deposited at The Cambridge Crystallographic Data Centre (CCDC) [40]; deposition number 1815949 [16].

**Table 5:** Summary of the pK_a_ values for the flavanones studied (the estimated standard deviations are given in parentheses).

Compound	Deprotonation Group	This Work		Literature Values
		Experimental	Theoretical	
6HF	6-OH	9.24 (±0.03)	13.43	9.90 [51]
	5′-OH chalcone	10.65 (±0.05)	15.05	–
7HF	7-OH	7.85 (±0.02)	8.28	7.26 [37]; 7.30 [51]
	4′-OH chalcone	10.78 (±0.04)	8.85	*ca*. 11.2 [37]
2′HF	2′-OH	10.90 (±0.04)	11.92	–
	2-OH chalcone	11.50 (±0.05)	9.86	–

**Table 6:** Spectra, K_b_ DNA intrinsic binding constants and the Wolfe-Shimer diagrams of flavanones (The plots were prepared with Origin(Pro) software using the original files from the instrument: Perkin-Elmer Lambda 11 spectrophotometer). Concentration of CT DNA (Tris) from 1.91 to 6.58 µM for 6HF, 2.3 to 33.2 µM for 7HF, and 4 to 10 µM for F and 2′HF.

Spectrum	Wolfe-Shimer Diagram	K_b_ [M^−1^]
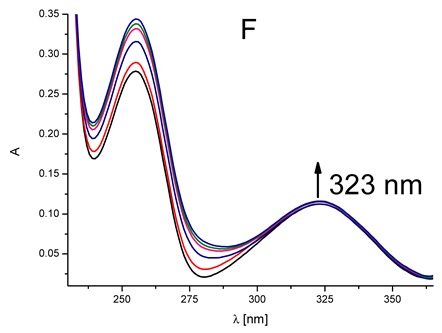	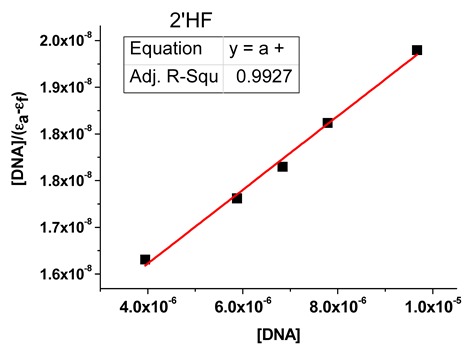	4.58 × 10^4^
**(flavanone)**		
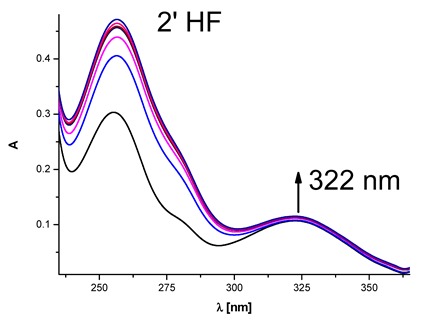	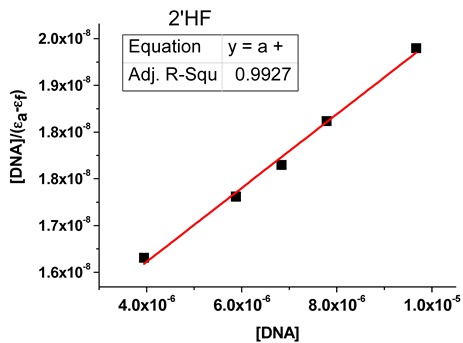	4.65 × 10^4^
(**2′-hydroxyflavanone**)		
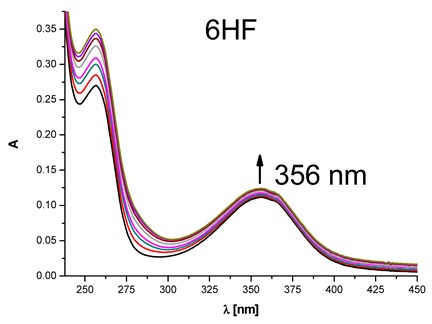	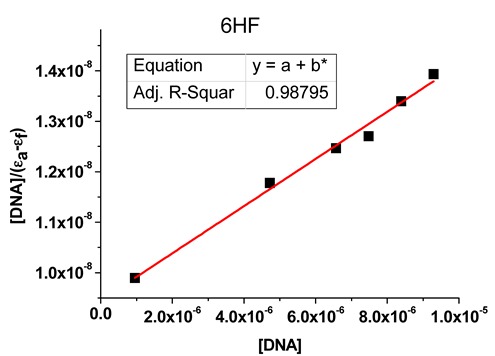	4 × 10^4^
(**6-hydroxyflavanone**)		
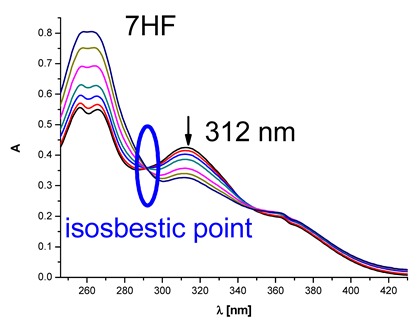	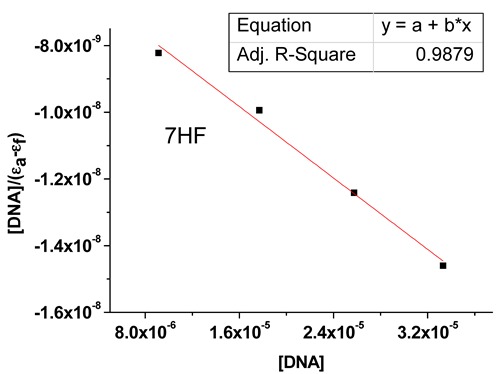	4.81 × 10^4^
(**7-hydroxyflavanone**)		

## Supplementary Materials

Figure S1: Absorption spectra of 2 × 10^−5^ M solutions of flavanone (F), 6-hydroxyflavanone (6-HF), 7-hydroxyflavone (7-HF) and 2′-hydroxyflavanone (2′-HF) at different pH. Figure S2: CV response of a 1.3*10-3 M solution of 6-HF in aqueous saline phosphate buffer, pH 7.4. Potential scan rate: 100 mV/s. Blue line: 1st scan; yellow line: 2nd scan; green line: 3rd scan. Figure S3: Titration curves obtained for monohydroxy flavanones: 2′-HF. 6-HF, 7-HF. Table S1: The relative stability of the two stereoisomers of the studied flavanones as predicted by the DFT(B3LYP)/6-31G(d,p)/PCM model. Table S2: The TD (n states = 10) DFT(B3LYP)/6-31+G(d,p)/PCM calculated spectroscopic parameters (transition electric dipole moment (μ); wavelength corresponding to the excitation energy (λ) and oscillator strength (f)) of the electronic transitions to the three low-lying excited singlet states in the studied chalcones. Table S3: Quantum chemical descriptors for chalcones derived from F, 2′HF, 6HF and 7HF (hardness (η); electronegativity (χ); chemical potential (μ); electrophilicity index (ω); softness (S)) calculated from ionization potential (IP) and electron affinity (EA) values, which were estimated by orbital vertical method.
