# Supplementary material for: Position Impact of Hydroxy Groups on Spectral, Acid–Base Profiles and DNA Interactions of Several Monohydroxy Flavanones
Source: Molecules. 2019 Aug 22;24(17):3049. doi: 10.3390/molecules24173049 (PMC6749416; doi:10.3390/molecules24173049)
Supplement: Supplementary file 1 [file molecules-24-03049-s001.pdf]

Position impact of hydroxyl group on spectral, acid-base profiles and DNA interactions of several monohydroxy flavanones

Elżbieta Łodyga-Chruścińska [1,*], Agnieszka Kowalska-Baron [1], Paulina Błazińska [1], Maria Pilo [2] , Antonio Zucca [2] , Violetta Korolevich [3] and Vitali T. Cheshchevik [3]

1 Faculty of Biotechnology and Food Sciences, Lodz University of Technology, Stefanowskiego Street 4/10, 90-924 Lodz, Poland; elalodyg@p.lodz.pl (E.Ł.C.); agnieszka.kowalska-baron@p.lodz.pl (A.K.B.); paulina.blazinska@edu.p.lodz.pl (P.B.)

2 Dipartimento di Chimica e Farmacia, Università di Sassari, via Vienna 2, I-07100 Sassari, Italy; mpilo@uniss.it (M.P.); zucca@uniss.it (A.Z.)

3 Polessky State University, Str. Dnieper flotilla 23, 225710 Pinsk, Republic of Belarus; Violetta.korolevich@mail.ru (V.K.); vitaliij@yandex.ru (V.T.C.)

* Correspondence: elalodyg@p.lodz.pl; Tel.: (+48-4263-134-17); (E.Ł.C.)

Supplementary Materials:

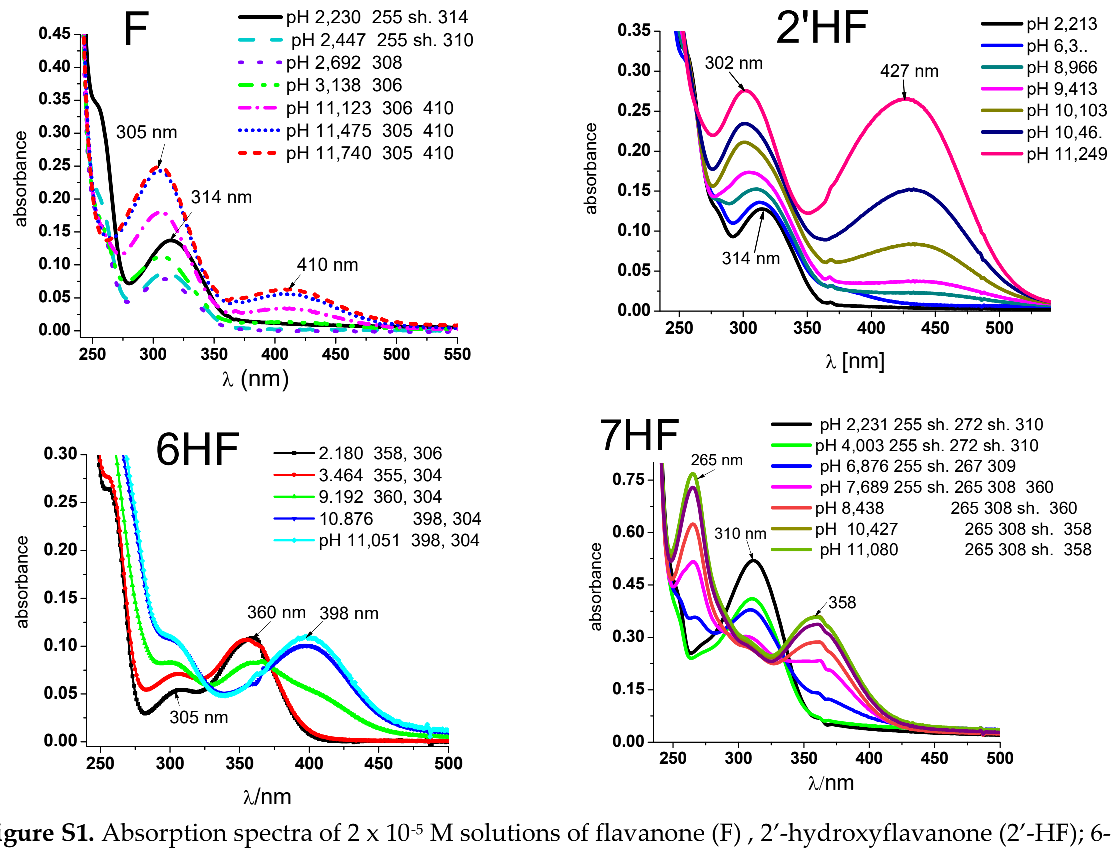

Figure S1. Absorption spectra of 2 x 10^{-5} M solutions of flavanone (F) , 2′-hydroxyflavanone (2′-HF); 6-hydroxyflavanone (6-HF), 7 and -hydroxyflavone (7-HF) at different pH.

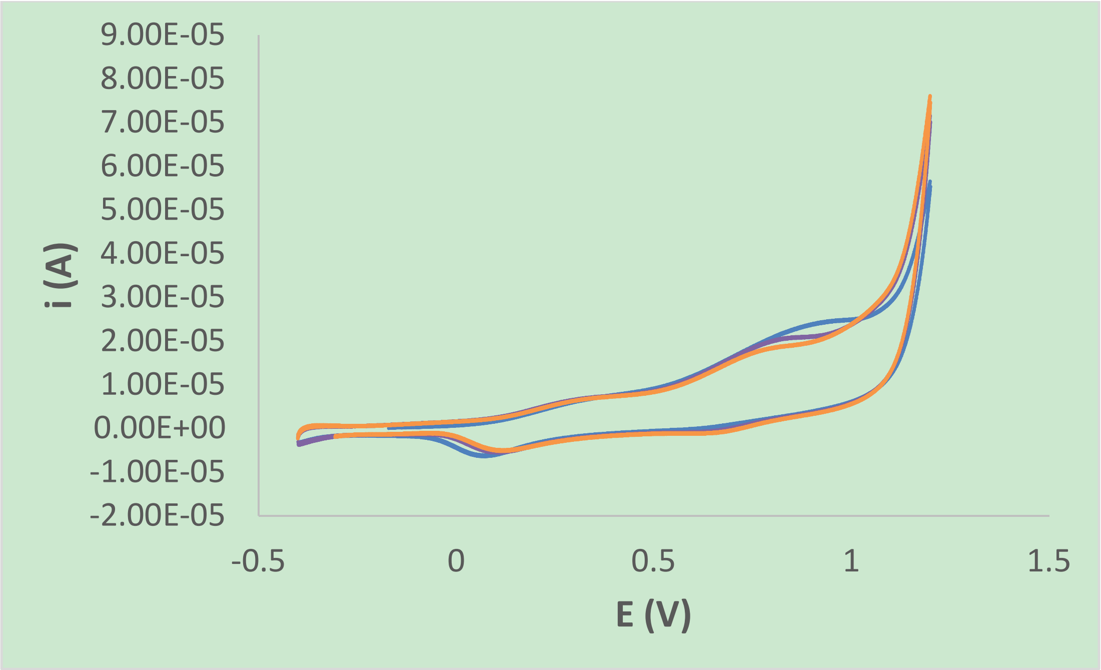

Figure. S2. CV response of a 1.3*10^{-3} M solution of 6-HF in aqueous saline phosphate buffer, pH 7.4. Potential scan rate: 100 mV/s. Blue line: 1st scan; yellow line: 2nd scan; green line: 3rd scan.

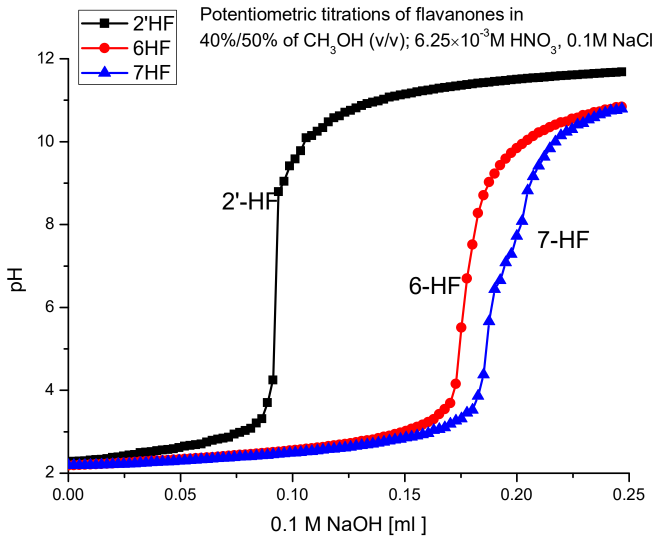

Figure S3. Titration curves obtained for monohydroxy flavanones: 2′- HF. 6-HF, 7- HF.

Table S1. The relative stability of the two stereoisomers of the studied flavanones as predicted by the DFT(B3LYP)/6-31G(d,p)/PCM model

Compound	E 2R [a.u]	E 2S [a.u]	ΔE [a.u]	ΔE [kcal/mol]
F	-729.2984158	-729.3019667	0.003551	2.228
2’HF	-804.5220575	-804.5256747	0.003617	2.270
6HF	-804.5199135	-804.5235356	0.003622	2.273
7HF	-804.5252153	-804.5287032	0.003488	2.189

Table S2. The TD (n states=10) DFT(B3LYP)/6-31+G(d,p)/PCM calculated spectroscopic parameters (transition electric dipole moment (μ); wavelength corresponding to the excitation energy (λ) and oscillator strength (f)) of the electronic transitions to the three low-lying excited singlet states in the studied chalcones

Compound	$S_0 \rightarrow S_1$			$S_0 \rightarrow S_2$			$S_0 \rightarrow S_3$		
	μ [D]	λ [nm]	f	μ [D]	λ [nm]	f	μ [D]	λ [nm]	f
2’ OH chalcone (from F)	4.5234	386.13	0.3558	6.8348	343.99	0.6035	0.2674	309.55	0.0262
2-OH chalcone (from 2’HF)	8.0939	395.68	0.6214	0.6889	370.01	0.0566	3.0529	330.34	0.2807
5’ OH chalcone (from 6HF)	2.8612	446.81	0.1945	8.5404	347.51	0.7465	0.2604	310.52	0.0255
4’ OH chalcone (from 7HF)	8.2473	375.17	0.6677	4.4210	338.28	0.3970	0.8058	309.78	0.0790

Table S3. Quantum chemical descriptors for chalcones derived from F, 2'HF, 6HF and 7HF (hardness (η); electronegativity (χ); chemical potential (μ); electrophilicity index (*ω*); softness (S)) calculated from ionization potential (IP) and electron affinity (EA) values, which were estimated by orbital vertical method.

Compound	E_{HOMO}* [eV]	E_{LUMO}* [eV]	HOMO-LUMO gap [eV]	IP [eV]	EA [eV]	η	χ	μ	*ω*	S
2' OH chalcone (from F)	-6.19	-2.43	3.76	6.19	2.43	1.88	4.31	-4.31	4.94	0.27
2-OH chalcone (from 2'HF)	-6.06	-2.38	3.67	6.06	2.38	1.84	4.22	-4.22	4.85	0.27
5' OH chalcone (from 6HF)	-5.69	-2.44	3.25	5.69	2.44	1.62	4.07	-4.07	5.09	0.31
4' OH chalcone (from 7HF)	-6.12	-2.31	3.80	6.12	2.31	1.90	4.22	-4.22	4.67	0.26

* calculated at the DFT(B3LYP)/6-31G(d,p)/PCM(water) method level of theory
